# Dietary Outcomes, Nutritional Status, and Household Water, Sanitation, and Hygiene (WASH) Practices

**DOI:** 10.1093/cdn/nzac020

**Published:** 2022-02-18

**Authors:** Oyedolapo Anyanwu, Shibani Ghosh, Meghan Kershaw, Abuye Cherinet, Eileen Kennedy

**Affiliations:** Tufts University, Friedman School of Nutrition Science and Policy, Boston, MA, USA; Tufts University, Friedman School of Nutrition Science and Policy, Boston, MA, USA; Tufts University, Friedman School of Nutrition Science and Policy, Boston, MA, USA; Save the Children, Addis Ababa, Ethiopia; Tufts University, Friedman School of Nutrition Science and Policy, Boston, MA, USA

**Keywords:** dietary diversity, WASH, MUAC, food groups, multisector, ENGINE

## Abstract

**Background:**

The Government of Ethiopia has made a major commitment toward improving food security, diet, nutrition, and health through a series of national nutrition plans. The focus of these plans is on providing both nutrition-specific as well as nutrition-sensitive approaches for achieving national priorities for health and nutrition. The present study conducted a secondary analysis of data provided through a larger birth cohort study conducted in Ethiopia between 2014 and 2016.

**Objectives:**

The overall objectives of this research were to assess the relation between minimum dietary diversity in women and water, sanitation, and hygiene (WASH), and evaluate the association between midupper arm circumference (MUAC) in women and WASH.

**Methods:**

In addition to descriptive statistics, the study used mixed effects logistic regression analyses to investigate the relation between dietary diversity, MUAC, and household WASH practices.

**Results:**

Improved WASH practices were associated with an increased probability (*p* = 0.04) that a woman would consume a diet with foods from 5 or more food groups. A beneficial effect was observed for improved WASH practices and a decrease in low MUAC. Improved household WASH practices were successful in contributing to improved dietary diversity in women as well as an improved MUAC.

**Conclusions:**

Interventions aimed at improving the diet and nutritional status of women during and after pregnancy should include relevant WASH components as essential elements in multisector nutrition programming.

## Teaser

In Ethiopia, improvements in water, sanitation, and hygiene significantly improved a woman's minimum dietary diversity and midupper arm circumference.

## Introduction

Global nutrition initiatives such as the Scaling Up Nutrition Movement (SUN) (1) and United States Agency for International Development, USAID Feed the Future (2) emphasize a multisector approach to improving diet, health, and nutrition, with a particular emphasis on the first 1000 d. Within this context, the Government of Ethiopia (GoE) has made a major commitment toward improving food security, diet, nutrition, and health. These commitments are evident in the National Nutrition Strategy, the series of National Nutrition Plans (NNP), and the Seqota Declaration, which vows to end stunting in children aged <2 y by 2030 (3). Within the nutrition portfolio of the GoE, there is a particular emphasis on vulnerable groups including pregnant and lactating women and children up to the age of 2 y – a group commonly referred to as the first 1000 d.

The NNP I and II (3) were based on the premise that both direct, specific approaches as well as nutrition-sensitive policies and programs are needed to improve nutrition in Ethiopia due to the myriad number of underlying drivers of unhealthy diets and poor nutritional status. Although some focused, nutrition-specific interventions (vitamin A supplementation) have demonstrated effectiveness (4–5), the GoE has employed a multisector strategy to achieve the nutrition objectives in their nutrition programs since research suggests that nutrition-specific interventions, by themselves, are often insufficient in achieving the goals for improving nutrition and health (4–5). Examples of nutrition-specific interventions include those that address the immediate causes of maternal, fetal, and preschooler malnutrition (5). These approaches can include improving maternal diets, improving prenatal access to health care, maternal education, micronutrient supplementation, and disease prevention, to name a few. Nutrition-sensitive interventions address the underlying determinants of health and nutrition and focus on sectors outside the health domain. These strategies can include nutrition-sensitive agriculture and improving the water, health, and sanitary environment of the household (6).

To that end, ENGINE (Empowering New Generations for Improved Nutrition and Economic Opportunities), a USAID-funded project, was implemented in Ethiopia from 2011 to 2016. ENGINE used a multisectoral approach to achieve the goals articulated in the NNP. Chief among the objectives of the NNP was improved diet quality, nutritional status, and health of women and preschool-aged children.

Many factors influence a woman's nutritional status, foremost among them is diet and nutritional status prior to pregnancy. An equally important predictor of women's nutritional status is the overall water, health, and sanitation environment faced by households (7).

The purpose of the present article is 2-fold: *1*) to assess the relation between minimum dietary diversity in women (MDD) and water, sanitation, and hygiene (WASH), and *2*) to evaluate the association between midupper arm circumference in women (MUAC) and WASH.

## Methods

As part of the ENGINE project, a longitudinal birth cohort study was implemented from 2014 to 2016 in 3 woredas (districts) of Oromiya, including Woliso, Goma, and Tiro Afeta. The study sites were entirely rural. The present article is based on secondary analysis of extant data derived from a more extensive birth cohort study; details on the protocol used are provided in a separate publication (7). The overall goal of the study was to assess the effects of a multisectoral strategy to improve health and nutrition among pregnant women and children under the age of 2 y. Pregnant women (*n *= 4680) were recruited using a rolling enrollment and surveillance approach. The women were identified by health workers with pregnancy being confirmed via a urine test. The age range of women was 15 to 50 y; estimated gestation at time of enrollment in the study was 12 to 32 wk. The data were collected every 3 mo starting in pregnancy, at birth, 3-, 6-, 9-, and 12-mo postpartum. Data on the household, the pregnant woman, and her child were collected throughout the duration of the study. The present article is limited to a discussion of women's MDD, MUAC, and household WASH practices. Data on study time points, household wealth and the education of the head of the household were used to control for potential confounding factors. Ethical approval was granted from the Institutional Review Board of Jimma University in Ethiopia (RPGC/264/2013) and Tufts University in the USA (Tufts Health Sciences Campus IRB reference number: 11088) before commencement of the study. Informed consent was obtained from the participants after a detailed explanation of the objectives of the study. Data was registered and stored in a secured server and access to the data was upon permission of the principal investigators with personal identifiers removed. During the study, women or infants who experienced health problems were referred to a nearby health facility for proper medical care.

Descriptive statistics were calculated for the mother and study child: mean, percentages (%) to characterize the distribution of mothers’ weight, weight status, and proportion of low MUAC, from birth of infants to 1 y. Weight status was derived using the BMI formula: kg/m^2^ and WHO BMI cut-offs: underweight ≤18.5;  normal weight = 18.5 to <25;  overweight and obese ≥25 to <30. Next, the distribution of the MDD was examined by the levels of household WASH practices; the MDD has been demonstrated to be a valuable proxy indicator for the nutrient adequacy of the diet (8). MDD was defined as having consumed 5 or more food groups (of a total of 10 food groups) in the past 24 h and is presented as a proportion of women who have met the MDD. Low MUAC was defined as MUAC <23 cm (4). The focus was on low MUAC rather than weight as an outcome of interest in this analysis because it is often considered a more valid indicator for undernutrition in pregnant women (7). Although our analyses are for the postpregnancy period, weight retention due to pregnancy could still bias the results.

To assess the WASH behaviors of households, data were combined on household water source and use, sanitation, and hygiene practices to develop a composite WASH score. The WASH score was stratified into quartiles of WASH practice: level 1 = poor WASH level; level 2 = fair WASH level; level 3 = good WASH level; and level 4 = very good WASH level (9). The household water source/use measured whether there was rainwater harvesting and storage of water separately in the household; the sanitation and hygiene component was based on the method of waste disposal including the presence of a garbage pit, trash discarded in the garden, bush or open burning, toilet/latrine use for defecation and urination, child handwashing following defecation, and mother's handwashing prior to food preparation and before serving a meal and the child eating.

The wealth index was derived from household data based on a principal component analysis that included housing conditions and availability of basic services like treated water and electricity; the index was then categorized into quartiles (7).

Finally, a mixed effects logistic regression analysis was used to investigate the relation between MDD and household WASH practices. Although data was collected for MDD and MUAC across all study time points (pregnancy, at birth, 3-, 6-, 9-, and 12-mo postpartum), household WASH variables were assessed only at 3 time points (pregnancy, 3- and 9-mo postpartum.). Thus, the analyses focused on the timepoints for which there was complete data for all variables of interest. To assess the relation between MDD and WASH, time point (TP) 1 was eliminated from the analyses and only data for 3-mo and 9-mo postpartum was used due to the methodological difference in capturing dietary intake at pregnancy (use of quantitative 24-h recall) relative to the other study time points where qualitative 24-h recalls were used (*n *= 7812). For the MUAC and WASH relation, data at 3 time points were used: pregnancy, 3-mo and 9-mo postpartum (*n *= 11,506).

## Results

### Distribution of mothers’ anthropometrics from birth of infant to 1 y (TP3 to TP7): weight, weight status, and MUAC

**Table 1** presents data on the mean weight of women from time point 3 (birth of study child) to time point 7 (1 y of age of study child). Maternal weight gain during pregnancy was not available; data on preconception weights were also not available and women entered the birth cohort study at varying points of time, making measurement of weight gain during pregnancy impractical. As noted, MUAC is considered a good proxy for nutritional status of women.

**TABLE 1:** Distribution of mothers’ mean weight from birth to 12 mo postpartum

Study time point (TP)	Months postpartum	No. of observations	Mean weight in kg + SD	Min	Max
TP3	Birth	4427	52.98 ± 6.59	31.33	86.22
TP4	3 mo	4193	51.16 ± 6.50	32.83	86.07
TP5	6 mo	4068	50.17 ± 5.36	31.52	83.43
TP6	9 mo	4002	49.45 ± 6.24	31.53	81.92
TP7	12 mo	3601	48.89 ± 6.12	31.63	80.65
Total		20,291	50.62 ± 6.54	31.33	86.22

As shown in Table 1, the mean weight of mothers, not surprisingly, was highest at time point 3 – birth of the infant. Maternal weight decreased progressively from birth through to 12-mo postpartum (end of the study). The declining weight across the time periods is due in part to loss of prenatal weight gain; however, as already indicated, the exact contribution of pregnancy weight gain to this weight loss cannot be ascertained.

**FIGURE 1:**
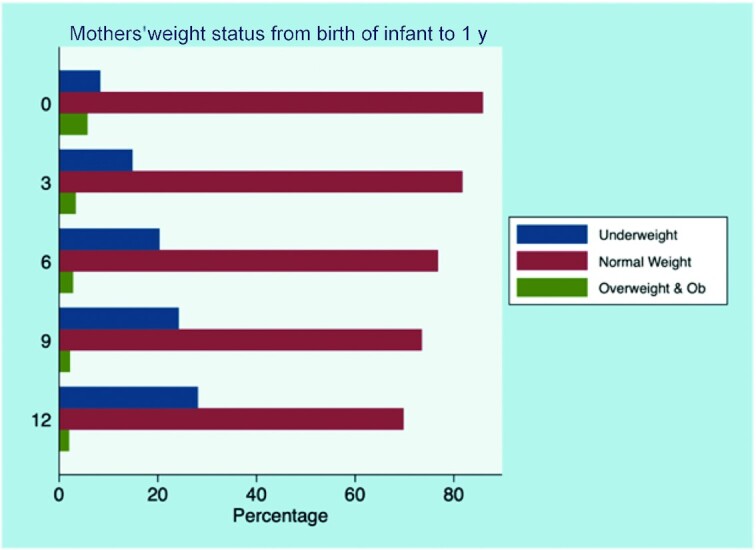
Mother's weight status over time. Ob, obese..

Most women have weights (**Figure 1**) within the normal range (BMI of 18.5 to 24.99). However, the proportion of women in the normal weight category steadily decreased from about 85% at birth of infant to 70% at 12-mo postpartum. Conversely, the proportion of underweight women increased from 8% at birth of the infant to about 28% at an infant age of 12 mo., a 20-percentage points difference. Overweight and obesity were less common in women.

**FIGURE 2:**
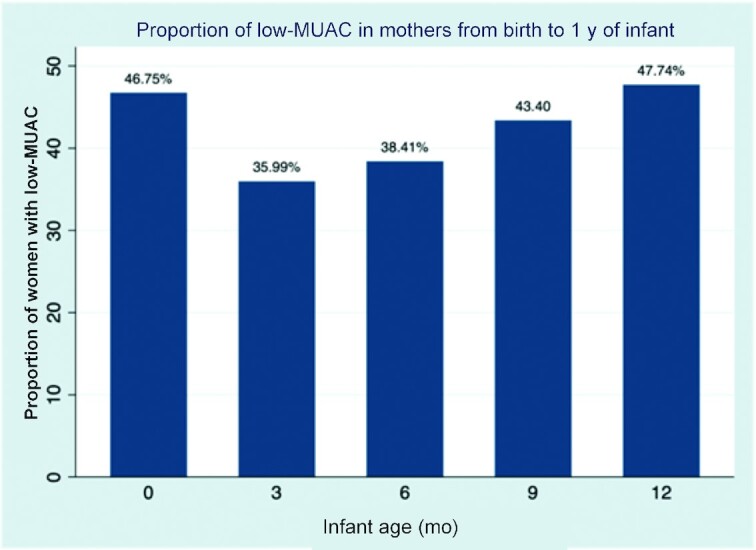
Proportion of low MUAC in mothers over time. MUAC, midupper arm circumference.

**Figure 2** shows the distribution of mothers with low MUAC from birth to 12-mo postpartum. The proportion of mothers with low MUAC markedly reduced from 46.75% at birth of infant to about 36% at infant age of 3 mo, but mothers became progressively thinner from infant age of 6 mo to 12 mo. This makes sense, because during the period of birth to the age of 3 mo, mothers typically have a better diet with higher intake of animal source foods and more diet diversity. The traditional diet of Ethiopian women in rural areas has limited diet variety, based primarily on a basic staple (10).

### Women's dietary outcome and household WASH practices

**Table 2** shows a positive relation between MDD and household WASH practice at infant ages of 3 to 6 mo. The proportion of women meeting the MDD increased dramatically from 8.8% to 21.5% in tandem with higher levels of household WASH practices (*P *<0.0001).

**TABLE 2:** Proportion of women meeting MDD by WASH practice levels at 3 and 9 mo postpartum combined

WASH practice levels (*n *= 8197)	No. of observations	MDD met yes (%)	MDD-met No (%)
1	2250	8.80	91.20
2	2888	13.30	86.70
3	1109	18.49	81.51
4	1950	21.54	78.46

Notes: WASH practice level created by summing WASH variables into a score, from 0 to 16, and then categorizing into quartiles of WASH practice: 1 = poor WASH Practice; 2 = fair WASH Practice; 3 = good WASH Practice; 4 = very good WASH Practice. MDD, minimum dietary diversity; MUAC, midupper arm circumference; WASH, water, sanitation, and hygiene.

**FIGURE 3:**
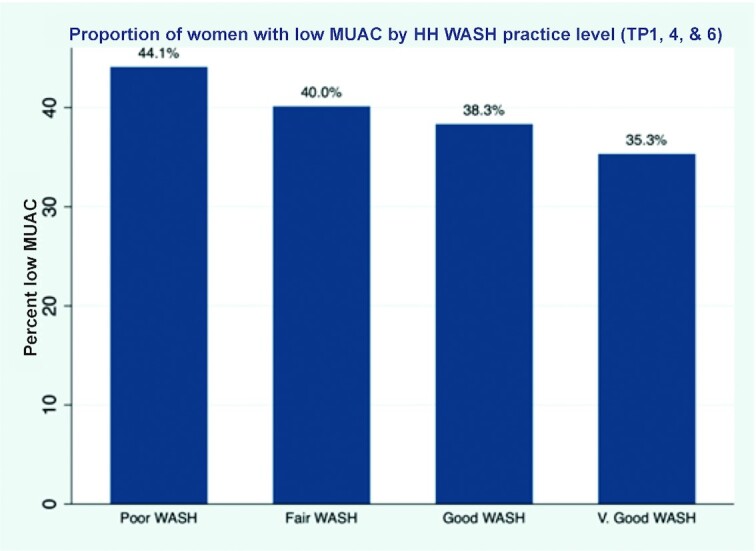
Low MUAC by HH WASH practice level in pregnancy, at 3 and 9 mo postpartum. HH, household; MUAC, midupper arm circumference; WASH, water, sanitation, and hygiene.

A similar beneficial effect was observed for improved household WASH behaviors on the proportion of mothers with low MUAC across 4 time points – the percentage of women with low MUAC decreased from 44.1% to 35.3% in the highest WASH category (**Figure 3**).

### Do household WASH behaviors significantly predict mother's dietary outcomes?

**Table 3** presents the results for the association between the proportion of women meeting MDD and WASH scores. The model in Table 3 was adjusted for study time point, wealth quintile, and education level of head of household. When the WASH score was divided into quartiles of WASH practices, the odds of achieving MDD were significant (*P* = 0.040). Compared with households with poor WASH scores, women from households in the fair WASH level had 24% increased odds of meeting the MDD for the good and the very good WASH levels, the odds of meeting the MDD increased by 45% and 41%, respectively.

**TABLE 3:** Association between MDD and WASH practice levels at 3–9 mo postpartum

Outcome var:	MDD met (yes/no) (Model 1: unadjusted univariate)			MDD met (yes/no) (Model 2: adjusted^1^)		
Wash practice	OR	95% CI	*P* value	OR	95% CI	*P* value
Wash score	1.08	1.103–1.14	0.001**	1.05	1.00–1.10	0.068
WASH practice quartiles						
1	Ref.	—	—	Ref.	—	—
2	1.31	1.06–1.62	0.012*	1.24	1.003–1.54	0.047*
3	1.60	1.23–2.09	0.001**	1.45	1.10–1.89	0.007*
4	1.69	1.30–2.20	0.0001***	1.41	1.08–1.85	0.012*
		*P*-trend	0.0007**		*P*-trend	0.040*

Notes: *n *= 7812. ^1^Model 2 adjusted for: study time point, wealth quintile, and household head education level. WASH practice quartiles: 1 = poor; 2 = fair; 3 = good; 4 = very good. *P* values: *indicates statistical significance at 5%, ** at 1%, and *** at 0.1% level of significance.

MDD, minimum dietary diversity; WASH, water, sanitation, and hygiene.

The association between low MUAC and the household WASH score was significant (*P* = 0.017) (**Table 4**). For every unit increase in the household WASH score, the odds of a low MUAC decreased. Compared to a household with a poor WASH score, households in the fair WASH category had 20% reduced odds of a low MUAC. The improvement in women's MUAC was a reduced odds of 29% and 25% for those in the good and very good categories, respectively.

**TABLE 4:** Association between low MUAC and WASH practice in pregnancy, at 3 and 9 mo postpartum

Outcome var:	Low MUAC (yes/no) (Model 1: unadjusted univariate)			Low MUAC (yes/no) (Model 2: adjusted^1^)		
Wash practice	OR	95% CI	*P* value	OR	95% CI	*P* value
Wash score	0.92	0.88–0.96	0.0001***	0.94	0.90–0.99	0.008*
WASH practice levels						
1	Ref.	—	—	Ref.	—	—
2	0.77	0.65–0.91	0.002*	0.80	0.68–0.95	0.009*
3	0.66	0.52–0.83	0.0001***	0.71	0.56–0.90	0.005*
4	0.67	0.53–0.84	0.001**	0.75	0.59–0.96	0.022*
		*P*-trend	0.0006**		*P*-trend	0.017*

Notes: *n *= 11,506. ^1^Model 2 adjusted for: study time points, wealth quintile, and household head education level. WASH practice quartiles: 1 = poor; 2 = fair; 3 = good; 4 = very good. *P* values: *indicates statistical significance at 5%, ** at 1%, and *** at 0.1% level of significance. MUAC, midupper arm circumference; WASH, water, sanitation, and hygiene.

## Discussion

The Sustainable Development Goals (SDGs) announced by the UN in 2015 (11) stress the availability of clean water and proper sanitation as critical elements for achieving SDG 6 – global health. In Ethiopia, of the total population, only 38% have access to a safe drinking water source and only 12% use improved sanitation facilities (12). Thus, the issue of lack of water and sanitation facilities is one concern of the GoE that is reflected in the multisector nutrition plans.

Much of the research on the effects of WASH interventions on nutrition have focused on preschool-aged children, and to a lesser extent, on pregnant and lactating women (13). There is clear evidence that effective WASH interventions reduce malnutrition levels by reducing enteric infections through improved water and sanitation in low- and middle-income countries (14).

The research on the effectiveness of individual components of WASH on decreasing malnutrition and/or improving nutritional status provides mixed results (13, 15–20). Several reasons account for this ambiguity; many studies have examined only certain components of WASH and not the range of practices in the entire domain of water and sanitation. The impact indicator in evaluating WASH varies, with most typically a measure of underweight, stunting, or rates of diarrhea in children used to assess impact. The research designs and length of WASH interventions have varied from several months to several years. These combined factors make it impossible to derive consensus on the most effective WASH policies and programs.

The data presented in this brief are one of the first instances in which the association between women's dietary diversity, MUAC, and household WASH practices have been documented. The results are encouraging. Both the MDD and MUAC in women improved as the household WASH scores improved. As noted, the odds of improved MDD increased anywhere from 24% to 45%. Similarly, the probability of a low MUAC within the WASH categories decreased from 20% to 29% as the WASH scores improved.

There are 3 components of any intervention that are important in assessing effectiveness: impact; feasibility/practicality; sustainability. The positive impact of improved WASH on the dietary outcomes of women is unambiguous from the results presented. Improved dietary diversity and nutritional status, as measured by MUAC, were evident as the quality of WASH at the household level improved. Thus, the positive impact of WASH practices has been demonstrated as part of the ENGINE multisector nutrition project.

This study used a composite indicator to reflect WASH; this included measures of water source and use, waste disposal methods, latrine/toilet use, and handwashing practices around cooking, preparing, and serving foods, as well as child feeding behaviors. Improvements in each of these components were feasible in the areas targeted by ENGINE.

Finally, the issue of sustainability of interventions is essential to ensure that the positive impacts are sustained. The investments in WASH infrastructure – improved water sources and harvesting, and improved sanitation facilities are likely to be sustained in the longer term. What is less clear is whether the behaviors that were promoted in handwashing techniques and hygienic practices related to child feeding will be similarly maintained. The evidence from successful behavior change campaigns has stressed the fact that these approaches must be regularly updated and revitalized to ensure long-term positive effects (13). Strategies to refresh communications around WASH activities should be a part of future nutrition plans in Ethiopia.

In conclusion, improved WASH activities were successful in contributing to improved dietary diversity in women as well as an improved MUAC. Interventions aimed at enhancing the diet and nutritional status of women during and after pregnancy should include relevant WASH components as essential elements in multisector nutrition programming.

## Data Availability

Data described in the manuscript, code book, and analytic code will be made available upon request pending publication of the manuscript.

## Notes

Author disclosures: The authors report no conflicts of interest.

Abbreviations used: ENGINE, Empowering New Generations for Improved Nutrition and Economic Opportunities; GoE, Government of Ethiopia; MDD, minimum dietary diversity; MUAC, midupper arm circumference; NNP, National Nutrition Program; SDG, Sustainable Development Goal; TP, time point; USAID, United States Agency for International Development; WASH, water, sanitation, and hygiene.
